# Evaluation of Remdesivir Utilization Pattern in Critically Ill Patients With COVID-19 in Jazan Province

**DOI:** 10.7759/cureus.36247

**Published:** 2023-03-16

**Authors:** Munif Ayyashi, Hussain Darbashi, Ahmed Hakami, Fahad Sharahili

**Affiliations:** 1 Pharmaceutical Care Department, Abu Arish General Hospital, Ministry of Health, Jazan, SAU; 2 Pharmaceutical Care Department, Jazan Health Affairs General Directorate, Ministry of Health, Jazan, SAU; 3 Pharmacy Department, Jazan University, Jazan, SAU

**Keywords:** hemoglobin, serum creatinine, ast, alt, covid-19, remdesivir

## Abstract

Background

Severe acute respiratory syndrome coronavirus 2 (SARS-CoV-2), the virus that causes coronavirus disease 2019 (COVID-19), has spread around the world, spurring the biomedical community to find and create antiviral therapies. The agent remdesivir, which has undergone a protracted and tortuous developmental path, is one potential therapeutic strategy now being assessed in several clinical trials. A broad-spectrum antiviral drug called remdesivir has already shown antiviral effects against filoviruses. Remdesivir was suggested as an exploratory medicine early in the pandemic because in vitro tests showed it to have antiviral effectiveness against SARS-CoV-2.

Methods

We conducted a retrospective cohort study that examined patient data captured through an electronic medical system at the Abu Arish General Hospital between 2021 and 2022. Data analysis was performed with SPSS version 25.0 (Armonk, NY: IBM Corp.).

Results

A total of 88 patients were included in this study. With the usage of remdesivir, our risk model is able to forecast adverse events and the case fatality rate. In contrast to D-dimer and c-reactive proteins, we showed that alanine transaminase (ALT), aspartate aminotransferase (AST), serum creatinine, and hemoglobin are relevant variables.

Conclusion

Our risk model can predict the adverse reactions and case fatality rate with the use of remdesivir. We demonstrated ALT, AST, serum creatinine, and hemoglobin as important variables rather than D-dimer and c-reactive proteins.

## Introduction

Severe acute respiratory syndrome coronavirus 2 (SARS-CoV-2), which causes the coronavirus disease 2019 (COVID-19), has high morbidity and mortality rates [1-3]. Risk factors for the development of acute respiratory distress syndrome and death include advanced age, male sex, neutrophilia, organ dysfunction, coagulopathy, and high D-dimer levels [4]. Due to the COVID-19 pandemic’s rapid evolution during the first wave, health authorities concentrated on repurposing already-approved medications to create timely and affordable therapeutic approaches [5,6]. The mainstay of medical care for hospitalized COVID-19 patients continues to be supportive care, which includes providing oxygen and administering dexamethasone to patients who have invasive mechanical ventilation [7]. Various medications were subject to expanded access programs (EAP) and emergency use authorization (EUA). EAP has enabled drug repositioning for COVID-19, allowing for the use of drugs, such as remdesivir, baricitinib, tocilizumab, nirmatrelvir-ritonavir, and molnupiravir, as well as early access to COVID-19 convalescent plasma, casirivimab-imdevimab, bamlanivimab/etesevimab, and sotrovimab [1,7-9]. Broad-spectrum action against members of various viral families, such as filoviruses, paramyxoviruses, and coronaviruses, has been demonstrated, and the drug has been shown to have both preventive and therapeutic effects against these coronaviruses in nonclinical animals [10-12]. Accordingly, remdesivir was advertised as a candidate medication for COVID-19 therapy [13-16].

The European Medicines Agency has prepared paperwork for compassionate use that summarizes the pharmacokinetics of remdesivir (EMA, 2020). Remdesivir is injected intravenously (IV) and is given in two doses: a loading dose on day 1 (200 mg in adults; pediatric patients receive a dose that is tailored for body weight) and a daily maintenance dose (100 mg in adults) for a maximum of 10 days. Remdesivir has been shown to have a short plasma half-life in nonhuman primates (t_1/2_ = 0.39 h), but intracellular levels of the triphosphate form are sustained after daily dosing of 10 mg/kg [17,18]. Remdesivir’s effectiveness against SARS-CoV-2 and related coronaviruses was supported by in vitro and preclinical in vivo animal models. Among these is a current in vitro investigation of remdesivir’s antiviral activity against SARS-CoV-2 (formerly known as 2019-nCov, strain nCoV-2019BetaCoV/Wuhan/WIV04/2019) that measured the viral copy number in infected Vero E6 cells using qRT-PCR. In this work, an IC50 of 770 nM and an IC90 of 1,760 nM (at cytotoxic concentrations > 100 mM) were found [19,20]. Remdesivir’s effectiveness in vivo in inhibiting viral replication and reducing viral-associated pathologies against related coronaviruses was further established in research by Sheahan et al. and de Wit et al. [11,21].

As mentioned above, remdesivir (GS-5734) is a phosphoramidate prodrug of a monophosphate nucleoside analog (GS-441524) that inhibits the replication of viral genomes by RNA-dependent RNA polymerase (RdRp) [22,23]. Nucleoside analogs are not thought to pass easily through the cell wall. After entering the host cell, they must undergo phosphorylation to form nucleoside triphosphate (NTP), which is similar to adenosine triphosphate (ATP) and can be utilized for genome replication by RdRp enzymes or complexes [24-26]. Remdesivir is metabolized by host cells into its pharmacologically active analog adenosine triphosphate (GS-443902), which then competes with ATP for integration into the nascent RNA strand by the RdRp complex. This results in the termination of RNA synthesis, which restricts viral replication after a few more nucleotides have been incorporated [23,24]. In primary human airway epithelial cultures and human lung cells, remdesivir showed strong antiviral efficacy against SARS-CoV-2. With a half-maximal effective concentration, remdesivir also has a dose-dependent inhibitory effect on SARS-CoV-2 replication (EC50) [27,28]. Remdesivir is a substrate for the cytochrome P450 (CYP450) enzymes CYP2C8, CYP2D6, and CYP3A4, as well as the organic anion-transporting polypeptide OATP1B1, OATP1B3, and P-glycoprotein (P-gp) transporters. Remdesivir and its metabolites are thought to be CYP inhibitors in vitro, but there is no proof that they induce CYP. The possibility of clinically significant drug-drug interactions (DDIs) may be constrained by the drug’s mode of administration and quick clearance. However, more clinical research is necessary to assess how remdesivir interacts with the cytochrome P450 system and to identify any potential drug-drug interactions [29,30].

Finding the incidence of remdesivir use in the Intensive Care Unit (ICU) at Abu Arish General Hospital was one of the study’s key goals. Other goals of this study included determining the baseline traits of patients at the beginning of remdesivir treatment and in patients following remdesivir therapy. This study also evaluated the clinical results after using remdesivir.

## Materials and methods

Retrospective data analysis was performed on all patients treated with remdesivir at Abu Arish General Hospital between 2021 and 2022. The ethics committee’s approval was required before the study could be carried out. Information was gathered from the individual hospitals’ medical records departments. This study was initiated after approval from the Jazan Health Ethics Committee (reference number: 22110), which granted a waiver of written informed consent.

All patients aged 18 years and over with critical illnesses whose doctors recommended remdesivir for COVID-19 between 2021 and 2022 were included. Critically ill was defined as patients with an oxygen saturation of 94% or needing additional oxygen or mechanical breathing. Patients under the age of 18 years were excluded, as were cases where remdesivir was prescribed for a condition other than COVID-19. Those who were not diagnosed with COVID-19 or not seriously unwell were also excluded.

The data was collected from the electronic medical system of the hospital in Jazan. The collected data comprised patient demographic data. Prior to the completion of the remdesivir course, baseline data on clinical features, such as a history of hypertension, dyslipidemia, hypothyroidism, kidney dysfunction, heart failure (HF), diabetes mellitus, and the levels of serum creatinine (SrCr) (mmol/L), hemoglobin, glucose, alanine transaminase (ALT), and aspartate aminotransferase (AST), were determined. Data were reported as median and ranges, means, and standard deviations (SDs) for continuous variables. A chi-square test for association and analysis of variance (ANOVA) for mean differences were applied accordingly. All analyses were done at 5% significance using SPSS version 25.0 (Armonk, NY: IBM Corp.).

## Results

**Figure 1:**
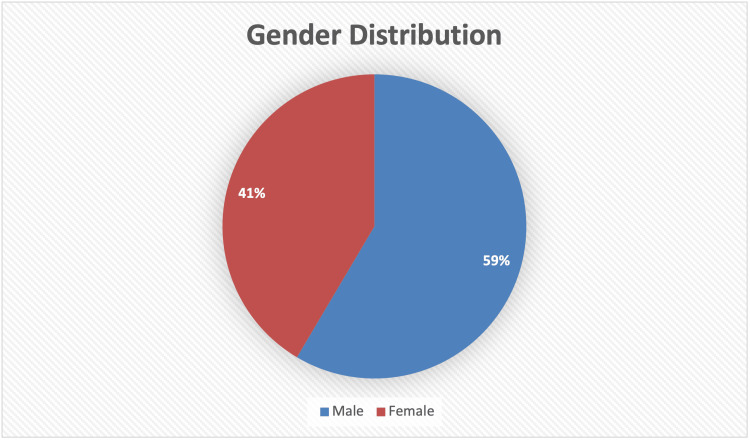
Graphical representation of the gender distribution of study population.

**Figure 2:**
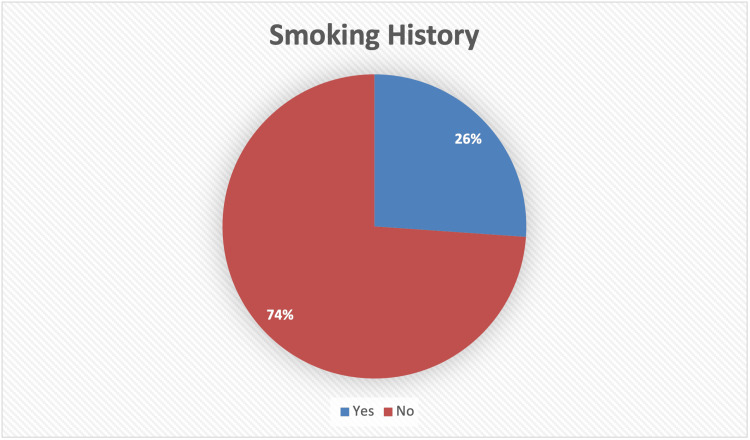
Graphical representation of the smoking history of the study population.

**Figure 3:**
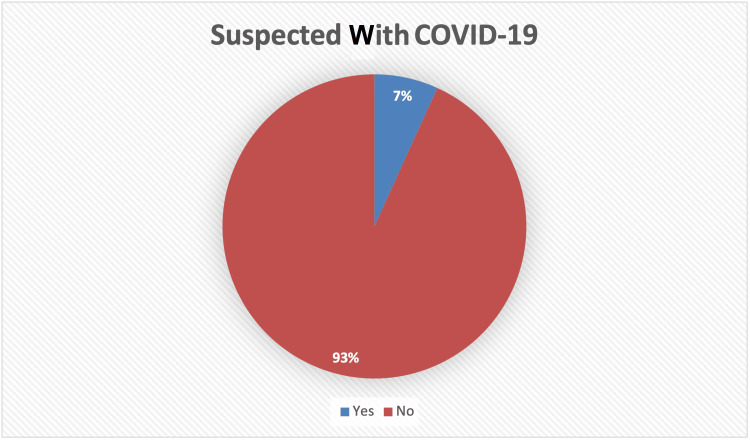
Graphical representation of the suspected participants with COVID-19 distribution. COVID-19: coronavirus disease 2019

**Figure 4:**
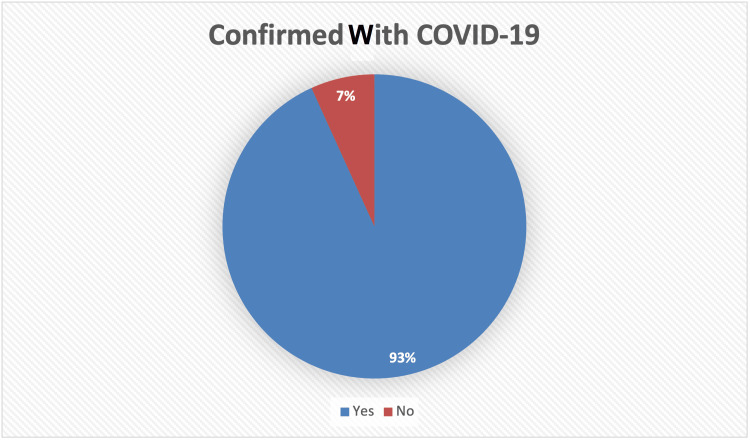
Graphical representation of the confirmed participants with COVID-19 distribution. COVID-19: coronavirus disease 2019

**Figure 5:**
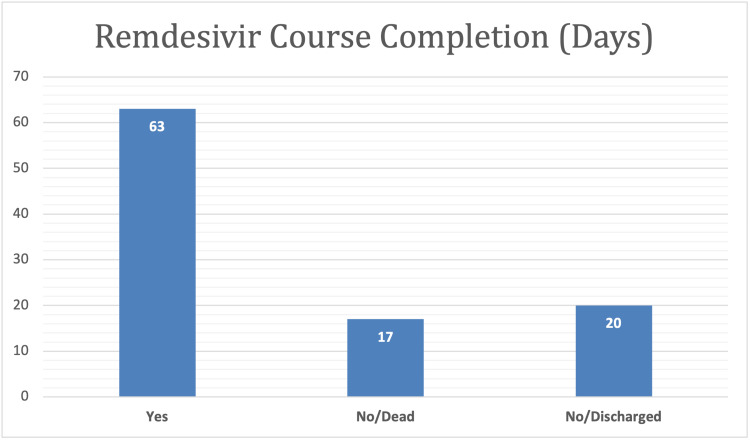
Graphical representation of the remdesivir course completion distribution in days.

**Figure 6:**
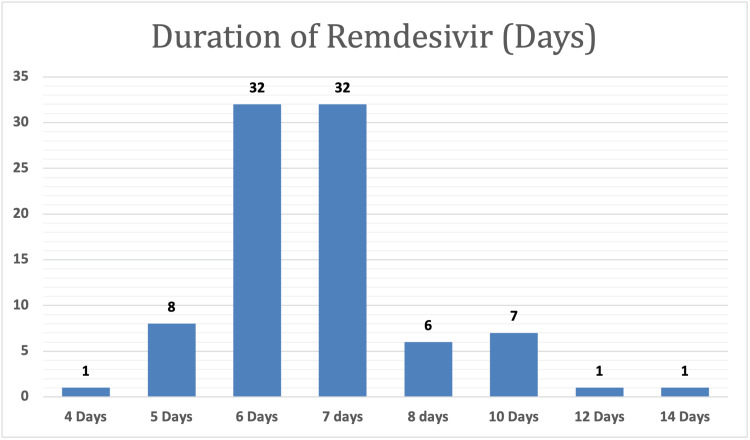
Graphical representation of the duration of remdesivir in days.

To explore the demographic and clinical characteristics of the study population, data were collected from a sample of 88 patients who had been administered “remdesivir” medicine, and the majority (93.2%) of them were confirmed to have COVID-19. Of these patients, 63% completed the course, 17% died, and 20% were discharged from the hospital. Furthermore, the results indicated that the majority of the patients were male (59.1%) and had used remdesivir for six to seven days (72.4%) (Table 1 and Figures 1-6).

**Table 1:** Demographic and clinical characteristics of the study population (N = 88). N is the total number of participants.

Variable	Level	N	%
Gender	Male	52	59.1
	Female	36	40.9
Suspected with COVID-19	Yes	6	6.8
	No	82	93.2
Confirmed with COVID-19	Yes	82	93.2
	No	6	6.8
Smoking history	Yes	23	26.1
	No	65	73.9
Remdesivir course completion	Yes	55	63.0
	No/dead	15	17.0
	No/discharged	18	20.0
Duration of remdesivir (days)	4	1	1.1
	5	8	9.1
	6	32	36.4
	7	32	36.4
	8	6	6.8
	10	7	8.0
	12	1	1.1
	14	1	1.1

Descriptive statistics, such as mean, standard deviation, minimum, and maximum, were calculated for the baseline data before the remdesivir course completion. Clinical characteristics included a history of hypertension, dyslipidemia, hypothyroidism, kidney dysfunction, HF, and diabetes mellitus; and levels of SrCr (mmol/L), hemoglobin, glucose, ALT, and AST (Table 2).

**Table 2:** Demographic and clinical characteristics before remdesivir course completion. BMI: body mass index; ALT: alanine transaminase; AST: aspartate aminotransferase; SrCr: serum creatinine; CrCl: creatinine clearance; SD: standard deviation

Variable	Mean	SD	Minimum	Maximum
Age	69.23	17.02	16.00	108.00
Weight	72.05	15.94	23.00	120.00
Height	164.94	5.90	146.00	188.00
BMI	26.41	5.48	10.79	44.92
ALT	47.18	88.48	6.20	663.00
AST	94.65	265.36	10.20	2378.00
Albumin	30.78	6.07	17.40	44.48
Bilirubin	15.73	18.82	2.90	124.30
Glucose	8.69	4.76	2.63	31.70
Hemoglobin	12.02	2.30	5.73	16.00
SrCr mmol/L	103.75	49.25	24.00	291.00
CrCl mL/min	66.16	37.63	20.00	256.00

The results regarding the remdesivir loading dose of 200 mg/day and the maintenance dose of 100 mg/day indicated all patients received the loading dose. However, 97.27% of the patients received the maintenance dose (Table 3).

**Table 3:** Descriptive statistics of remdesivir loading and maintenance dose per day. N: total number; SD: standard deviation; COVID-19: coronavirus disease 2019

Remdesivir	COVID-19 confirmed	N	Mean	SD	Minimum	Maximum
Loading dose 200 mg/day	Yes	82	198.78	11.04	100.00	200.00
	No	6	200.00	0.00	200.00	200.00
Maintenance dose 100 mg/day	Yes	80	100.00	0.00	100.00	100.00
	No	6	100.00	0.00	100.00	100.00

**Figure 7:**
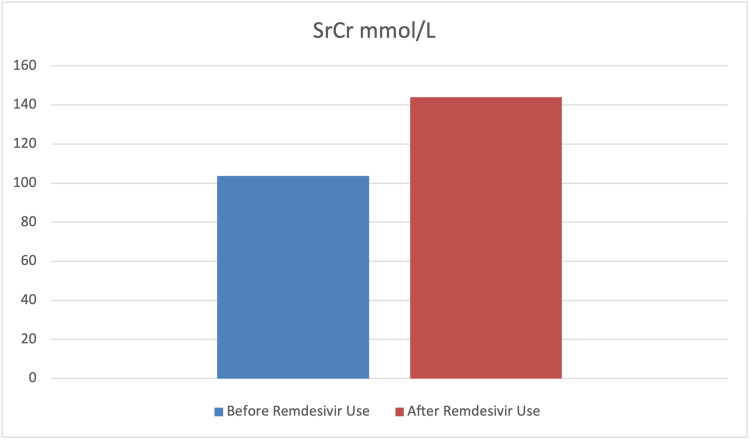
Graphical representation of the level of SrCr mmol/L before and after remdesivir use. SrCr: serum creatinine

**Figure 8:**
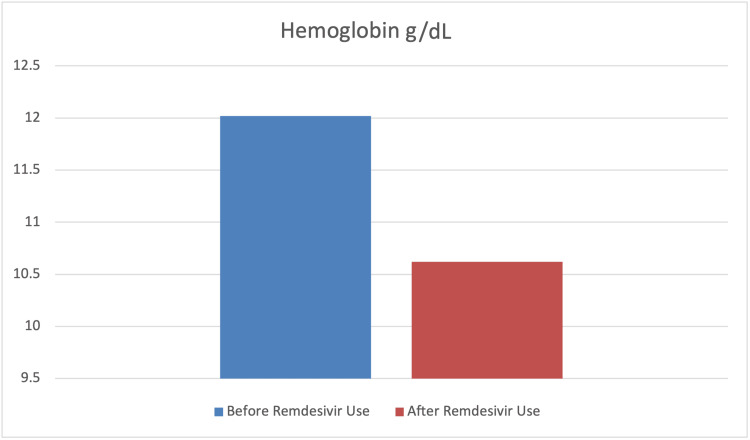
Graphical representation of the level of hemoglobin g/dL before and after remdesivir use.

**Figure 9:**
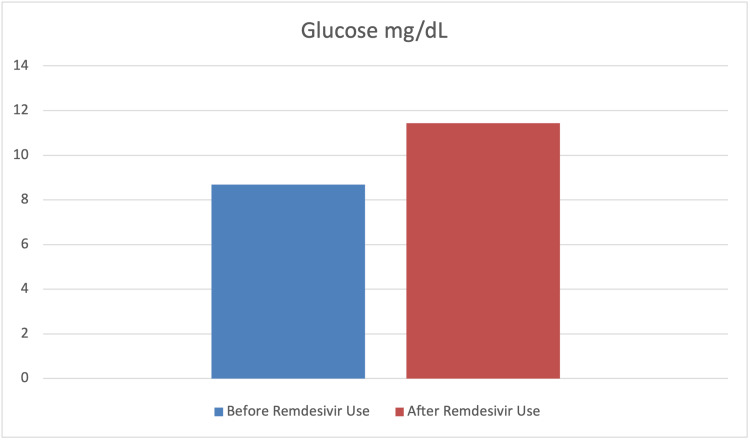
Graphical representation of the level of glucose mg/dL before and after remdesivir use.

**Figure 10:**
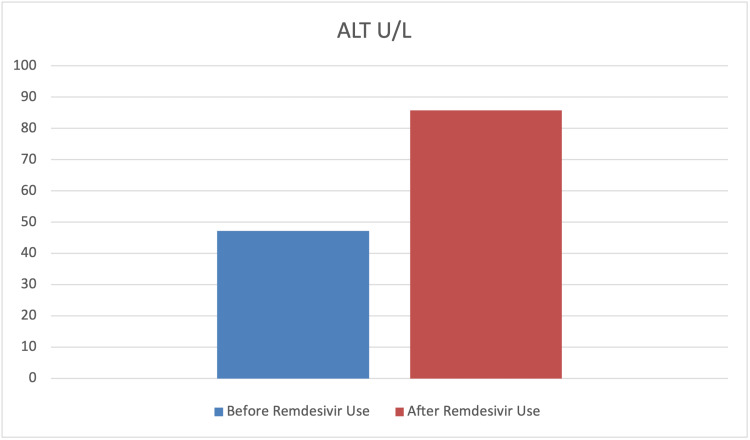
Graphical representation of the level of ALT U/L before and after remdesivir use. ALT: alanine transaminase

**Figure 11:**
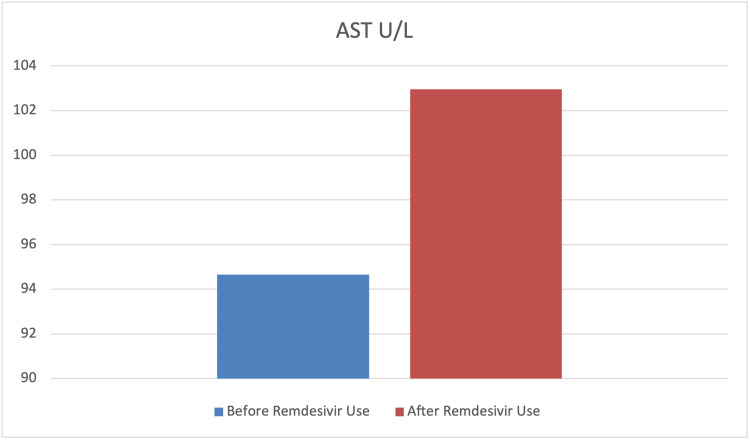
Graphical representation of the level of AST U/L before and after remdesivir use. AST: aspartate aminotransferase

To assess the effect of remdesivir use on the levels of SrCr, hemoglobin, glucose, ALT, and AST paired sample t-tests were carried out. The findings indicated a significant mean difference before and after remdesivir use in terms of SrCr, hemoglobin, glucose, and ALT levels (p<0.001). However, the mean difference before and after remdesivir use in terms of AST was not statistically significant (p>0.05). Furthermore, the results indicated that the levels of SrCr, glucose, and ALT had significantly increased after the use of remdesivir. In contrast, the level of hemoglobin significantly decreased after its use. The findings also indicated that although the level of AST increased after the use of remdesivir, this difference was not statistically significant (p>0.05) (Table 4 and Figures 7-11).

**Table 4:** Mean comparison before and after remdesivir use in SrCr, hemoglobin, glucose, ALT, and AST levels. ALT: alanine transaminase; AST: aspartate aminotransferase; SrCr: serum creatinine; SD: standard deviation; t: total number; SD: standard deviation; COVID-19: coronavirus disease 2019

Variable	Before remdesivir use		After remdesivir use		t (87)	p-Value	Cohen’s syndrome
	Mean	SD	Mean	SD			
SrCr mmol/L	103.75	49.25	143.91	103.39	-4.22	<0.001	0.45
Hemoglobin	12.02	2.30	10.62	2.07	9.92	<0.001	1.06
Glucose	8.69	4.76	11.43	6.18	-5.84	<0.001	0.62
ALT	47.18	88.48	85.77	143.76	-3.05	0.003	0.33
AST	94.65	265.36	102.95	140.21	-0.31	0.378	0.03

**Figure 12:**
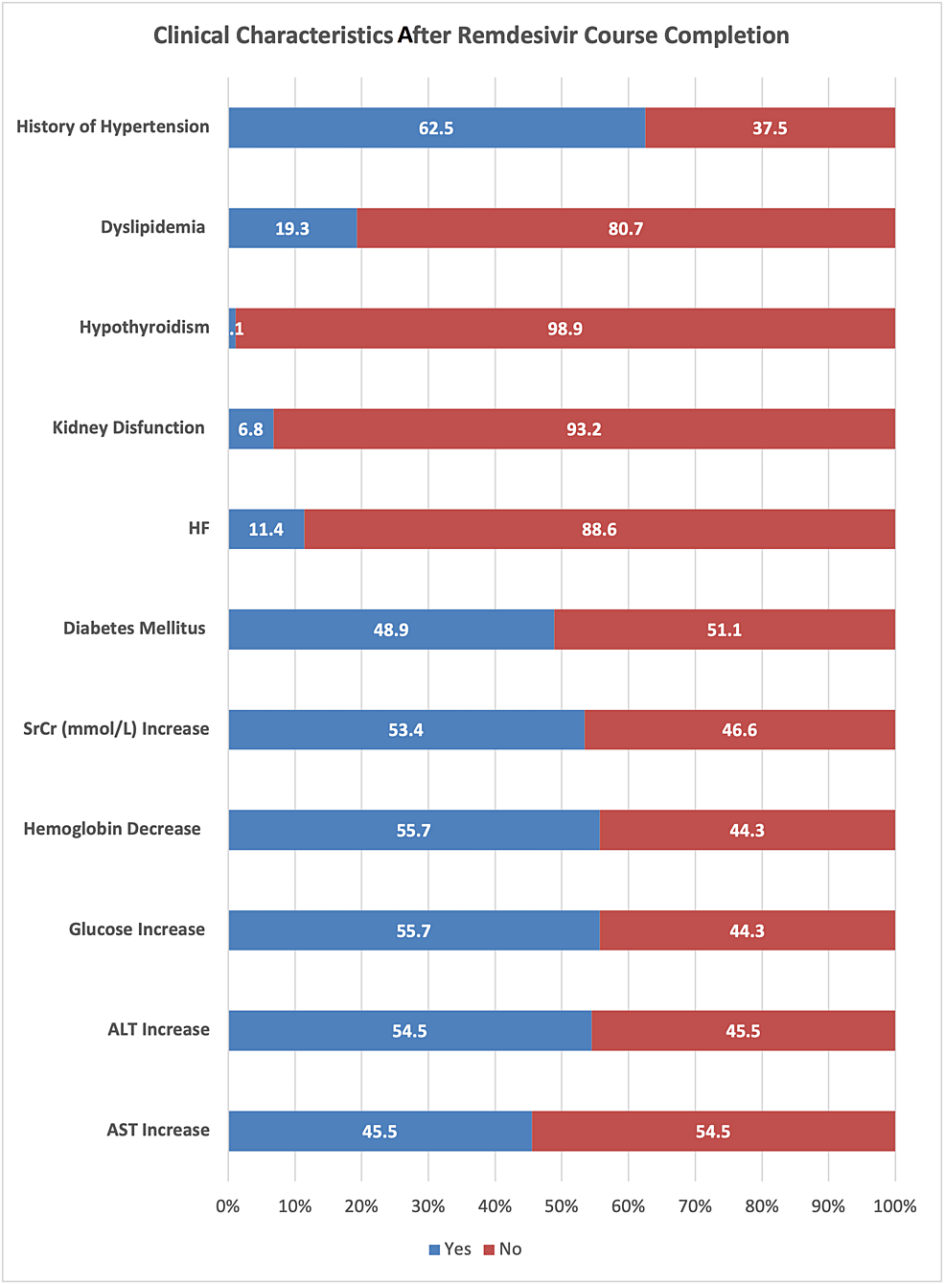
Graphical representation of the clinical characteristics after remdesivir course completion. HF: heart failure; ALT: alanine transaminase; AST: aspartate aminotransferase; SrCr: serum creatinine

When examining the clinical characteristics after administering remdesivir, the results indicated that a history of hypertension was found in more than 62% of the patients. However, the histories of dyslipidemia (19.3%), hypothyroidism (1.1%), kidney dysfunction (6.8%), and HF (11.4%) were found to be very low. Furthermore, the findings indicated that after administering remdesivir, increases in the levels of SrCr (mmol/L), glucose, ALT, and AST were noted in 53.4%, 55.7%, 54.5%, and 45.5% of the patients, respectively. However, the hemoglobin level decreased in more than 55% of the patients (Table 5 and Figure 12).

**Table 5:** Clinical characteristics after remdesivir course completion (N = 88). HF: heart failure; ALT: alanine transaminase; AST: aspartate aminotransferase; SrCr: serum creatinine; N: total participants

Variable	Level	N	%
History of hypertension	Yes	55	62.5
	No	33	37.5
Dyslipidemia	Yes	17	19.3
	No	71	80.7
Hypothyroidism	Yes	1	1.1
	No	87	98.9
Kidney dysfunction	Yes	6	6.8
	No	82	93.2
HF	Yes	10	11.4
	No	78	88.6
Diabetes mellitus	Yes	43	48.9
	No	45	51.1
SrCr (mmol/L) increase	Yes	47	53.4
	No	41	46.6
Hemoglobin decrease	Yes	49	55.7
	No	39	44.3
Glucose increase	Yes	49	55.7
	No	39	44.3
ALT increase	Yes	48	54.5
	No	40	45.5
AST increase	Yes	40	45.5
	No	48	54.5

## Discussion

At present, no specific antiviral drug is available for SARS-CoV-2 infection. Remdesivir, an adenosine analog developed to work specifically against the Ebola virus in 2014, is used on a compassionate basis in moderate-to-severely infected COVID-19 patients due to the lack of any other proven antiviral. Remdesivir is normally given for 5-10 days, which may be extended in severely ill patients, along with steroids. In our study, 63% of the patients received a complete course of remdesivir, 17% died, and 20% were discharged during the treatment course.

SARS-CoV-2 infection predominately involves the host’s respiratory system; however, SARS-CoV-2-induced renal and hepatic damage has also been reported [31,32]. Major pathophysiological features of liver injury in SARS-CoV-2 infection include hepatic lobular and portal inflammation, steatosis, congestion, focal necrosis with neutrophil infiltration, microthrombosis, and mild-to-moderate increases in liver aminotransferase (five times the upper limit of normal {ULN}) [33]. Remdesivir use in SARS-CoV-2 patients is associated with mild-to-moderate self-limiting hepatic injury, with no signs of jaundice [34]. Our study reported a statistically significant increase (p=0.003) in ALT in 54.5% of patients, while there were non-significant increases in AST seen in 45.5% of COVID-19-recovered patients after using remdesivir. Our results were almost similar to earlier studies, which have also reported mild-to-moderately elevated aminotransferase levels in 10-60% of COVID-19 patients after parenteral use of remdesivir with yet unknown mechanisms [1,35-37]. In our study, ALT was more increased than AST, which is contrary to some of the results published [38] but similar to others [39]. This self-limiting slight increase could be associated with the virus itself (as higher ACE2 expression on the hepatocyte’s surface), vascular endothelial injury and hypoxia [40], systemic inflammation [41], and drug-induced liver injury (DILI) [42]. Possible mechanisms of DILI include insulin resistance, lipid dystrophy, oxidative stress, and the inhibition of mitochondrial RNA polymerase. Remdesivir is metabolized in the liver by OATP1B and CYP3A4, which indicates the susceptibility of hepatocytes to drug-induced liver damage [43]. More than a 10 ULN increase in liver enzymes is alarming and should be addressed with great caution.

Hospitalized COVID-19 patients requiring intensive care may develop hyperglycemia (an increase in blood glucose {BG}), mainly due to increased glucose production, virus-induced immune response, and insulin resistance [44]. Moreover, direct SARS-CoV-2 damage to ACE3 BG receptors in pancreatic islets can cause hyperglycemia, even in non-diabetic COVID-19 patients. Acute-phase hyperglycemia could increase ACE2 expression, resulting in more viral particles entering host cells and delaying the recovery phase. Due to transient damage to beta cells, hyperglycemia may persist for three years after recovery from COVID-19 and is generally associated with poor outcomes of the disease [45,46]. As remdesivir is usually administered in combination with steroids, hyperglycemia is considered an indirect adverse drug reaction [47]. Our study reported increased BG levels in 55.7% of patients, which were statistically significant (p<0.001). These findings are similar to those reported earlier by Beigel et al., Awad et al., Hajjar et al., and Shrestha et al. [48-51].

Remdesivir is associated with acute kidney injury (AKI), an important complication in COVID-19 patients associated with a poor prognosis of disease, and AKI itself is associated with a 1.5-fold increase in serum creatinine levels from baseline [52]. Our study showed a statistically significant (p<0.001) increase in serum creatine levels in 53.4% of patients, which is similar to the findings reported by Kuno et al. and Sedighi et al., but contrary to those of Wong et al. [53-55].

Our study reported a significant decrease (p<0.001) in blood hemoglobin (Hb) levels in 55.7% of patients, which is similar to other findings in the literature [56,57]. The use of remdesivir is associated with a 2.8-fold risk of AKI [58]. Another study reported that SARS-CoV-2 can bind to the beta chain of hemoglobin through its surface glycoproteins, and their inhibition causes blood complications [59]. Elsewhere, SARS-CoV-2's ability to capture porphyrin has been examined, which in turn can inhibit heme metabolism. The interaction of SARS-CoV-2 with hemoglobin through ACE2, clusters of differentiation (CD)26, and CD147 has already been confirmed [60]. Decreased blood hemoglobin levels may result in sideroblastic anemia-like possibilities, along with myelodysplastic features and the need to replace worn-out erythrocytes. Red cell distribution width (RDW) is a marker of myelodysplasias, and COVID-19 data shows increased RDW in severely ill patients.

Our risk model allowed us to predict the adverse reactions and case fatality rates with the use of remdesivir. We demonstrated ALT, AST, serum creatinine, and hemoglobin as important variables rather than D-dimer and c-reactive proteins. The small sample size and retrospective nature of the analysis are the main limitations of this study.

## Conclusions

The scientific community has rallied to sufficiently discover and assess new medicines and vaccinations as the COVID-19 pandemic continues to spread across the globe, and this community includes academia, government laboratories, small biotechnology firms, and global pharmaceutical corporations. The quickest therapeutic approach to stopping the pandemic’s spread involves repurposing or repositioning an efficient small-molecule drug. Remdesivir, one of these prospective treatments, has proven effective against coronaviruses in both in vitro and in vivo settings.
